# Two Small Molecules Restore Stability to a Subpopulation of the Cystic Fibrosis Transmembrane Conductance Regulator with the Predominant Disease-causing Mutation*

**DOI:** 10.1074/jbc.M116.751537

**Published:** 2017-01-13

**Authors:** Xin Meng, Yiting Wang, Xiaomeng Wang, Joe A. Wrennall, Tracy L. Rimington, Hongyu Li, Zhiwei Cai, Robert C. Ford, David N. Sheppard

**Affiliations:** From the ‡ Faculty of Biology, Medicine and Health, School of Biological Sciences, University of Manchester, Michael Smith Building, Oxford Road, Manchester M13 9PL, United Kingdom and; the § School of Physiology, Pharmacology, and Neuroscience, University of Bristol, Biomedical Sciences Building, University Walk, Bristol BS8 1TD, United Kingdom

**Keywords:** ABC transporter, chloride channel, cystic fibrosis, cystic fibrosis transmembrane conductance regulator (CFTR), protein purification, protein stability, ivacaftor, lumacaftor

## Abstract

Cystic fibrosis (CF) is caused by mutations that disrupt the plasma membrane expression, stability, and function of the cystic fibrosis transmembrane conductance regulator (CFTR) Cl^−^ channel. Two small molecules, the CFTR corrector lumacaftor and the potentiator ivacaftor, are now used clinically to treat CF, although some studies suggest that they have counteracting effects on CFTR stability. Here, we investigated the impact of these compounds on the instability of F508del-CFTR, the most common CF mutation. To study individual CFTR Cl^−^ channels, we performed single-channel recording, whereas to assess entire CFTR populations, we used purified CFTR proteins and macroscopic CFTR Cl^−^ currents. At 37 °C, low temperature-rescued F508del-CFTR more rapidly lost function in cell-free membrane patches and showed altered channel gating and current flow through open channels. Compared with purified wild-type CFTR, the full-length F508del-CFTR was about 10 °C less thermostable. Lumacaftor partially stabilized purified full-length F508del-CFTR and slightly delayed deactivation of individual F508del-CFTR Cl^−^ channels. By contrast, ivacaftor further destabilized full-length F508del-CFTR and accelerated channel deactivation. Chronic (prolonged) co-incubation of F508del-CFTR-expressing cells with lumacaftor and ivacaftor deactivated macroscopic F508del-CFTR Cl^−^ currents. However, at the single-channel level, chronic co-incubation greatly increased F508del-CFTR channel activity and temporal stability in most, but not all, cell-free membrane patches. We conclude that chronic lumacaftor and ivacaftor co-treatment restores stability in a small subpopulation of F508del-CFTR Cl^−^ channels but that the majority remain destabilized. A fuller understanding of these effects and the characterization of the small F508del-CFTR subpopulation might be crucial for CF therapy development.

## Introduction

Cystic fibrosis (CF)^8^ is a common life-shortening inherited disease, mostly affecting people of European origin (1). The disease affects multiple organ systems throughout the body, especially the respiratory airways and intestine, leading to the blockage of ducts and tubes by thick tenacious mucus and a failure of host defense systems (1, 2). CF is caused by mutations in the cystic fibrosis transmembrane conductance regulator (CFTR) (3), a unique ATP-binding cassette (ABC) transporter that functions as a Cl^−^ channel with complex regulation (4). Located in the apical membrane of epithelia, CFTR plays a pivotal role in transepithelial ion transport, regulating the quantity and composition of epithelial secretions (5).

To date, >2,000 mutations have been identified in the *CFTR* gene (see the Hospital for Sick Children in Toronto Cystic Fibrosis Mutation Database), although the vast majority are very rare and not all lead to CF (1). By far the most common disease-causing mutation, with a prevalence as high as 90%, is F508del, the deletion of the phenylalanine residue at position 508 of the CFTR amino acid sequence (1). F508del affects a residue located in a crucial position on the surface of the first nucleotide-binding domain (NBD1) (6). Structural studies reveal that the absence of Phe-508 causes local structural changes to the ABC α-subdomain of NBD1, mainly affecting the loop spanning residues 509–511 (7). However, loss of Phe-508 disrupts domain-domain interactions critical for correct assembly and function of CFTR (8–11). F508del-CFTR is recognized as abnormal by the endoplasmic reticulum quality control machinery and is ubiquitinated and degraded by the proteasome (12, 13). Thus, F508del principally disrupts the processing and intracellular transport of CFTR protein. Nevertheless, any F508del-CFTR that escapes to the plasma membrane exhibits two further abnormalities: reduced stability (14) and altered channel gating (15).

To overcome the defective expression and function of CF mutants, two classes of small molecules have been developed termed CFTR correctors and CFTR potentiators (12). CFTR correctors (*e.g.* lumacaftor; VX-809) (16) traffic mutant protein to the plasma membrane. By contrast, CFTR potentiators (*e.g.* ivacaftor; VX-770) (17) strongly augment channel gating. Although neither lumacaftor nor ivacaftor, by themselves, have clinical benefit for CF patients with the F508del mutation (18, 19), combination therapy with the two drugs has improved lung function and disease stability (20), leading to the approval in 2015 of lumacaftor-ivacaftor combination therapy.

When compared with the clinical benefit achieved by ivacaftor in CF patients with the gating mutation G551D (21), that observed with lumacaftor-ivacaftor combination therapy in CF patients homozygous for F508del appears less marked (20). One potential explanation of these data is that lumacaftor only partially rescues the plasma membrane expression of F508del-CFTR; combinations of correctors will probably be required for complete rescue (22, 23). However, chronic treatment of F508del-CFTR-expressing cells with ivacaftor destabilizes lumacaftor-rescued F508del-CFTR Cl^−^ currents (24, 25). Although this result argues that ivacaftor might have counterproductive effects on F508del-CFTR, other studies offer alternative explanations, including limited efficacy of lumacaftor (26) and the action of lumacaftor-ivacaftor combination therapy on mucociliary clearance (27).

Thus, the aims of this study were 2-fold: first, to understand better the impact of F508del on CFTR stability, and second, to investigate the effects of lumacaftor and ivacaftor on the instability of F508del-CFTR. To address our aims, we used biochemical and functional assays, purified CFTR protein, and recombinant CFTR expressed in cultured cells. We discovered that the F508del mutation destabilized purified full-length CFTR protein by ∼10 °C and deactivated CFTR Cl^−^ channels in excised membrane patches by the time-dependent loss of both channel gating and current flow through open channels. We found that lumacaftor and ivacaftor had complex effects on F508del-CFTR. Whereas lumacaftor thermally stabilized purified full-length F508del-CFTR protein, ivacaftor destabilized it. At the macroscopic level, chronic ivacaftor treatment accelerated the temporal deactivation of lumacaftor-rescued F508del-CFTR Cl^−^ currents. However, at the single-channel level, chronic lumacaftor and ivacaftor co-treatment greatly increased F508del-CFTR channel activity and stability in most, but not all, excised membrane patches. We conclude that lumacaftor-ivacaftor combination therapy rescues a small subpopulation of F508del-CFTR Cl^−^ channels. We speculate that understanding the heterogeneity of CFTR populations in epithelial cells might be crucial for CF therapy development.

## Results

### 

#### 

##### F508del, but Not G551D, Destabilizes CFTR Channel Activity in Cell-free Membrane Patches at 37 °C

Here, we investigated the instability of the most common CF mutant F508del-CFTR and its rescue by the CFTR corrector lumacaftor (16) and the CFTR potentiator ivacaftor (17). We analyzed the functional instability of F508del-CFTR in excised membrane patches and polarized epithelia at 37 °C to relate molecular defects to tissue dysfunction. To complement these studies, we examined the thermal instability of full-length F508del-CFTR protein in membranes and after purification. As controls, we studied wild-type CFTR and the CF mutation G551D, which severely disrupts CFTR channel gating without altering protein processing and stability (28–30).

**FIGURE 1.**
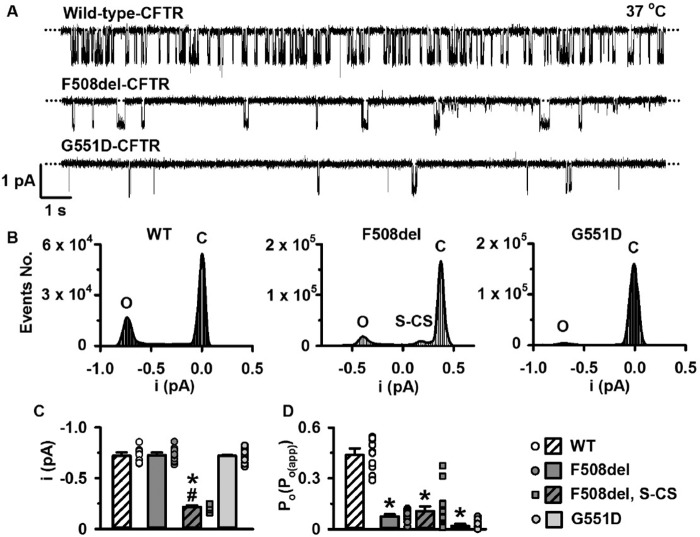
**The single-channel activity of wild-type CFTR, F508del-CFTR, and G551D-CFTR.** *A*, representative CFTR single-channel recordings in excised inside-out membrane patches from BHK cells expressing wild-type and F508del-CFTR and FRT cells expressing G551D-CFTR. In this and subsequent figures using the patch clamp technique, ATP (1 mm) and PKA (75 nm) were continuously present in the intracellular solution, temperature was 37 °C, voltage was −50 mV, and there was a large Cl^−^ concentration gradient across the membrane patch (internal [Cl^−^], 147 mm; external [Cl^−^], 10 mm). The *dotted lines* indicate where channels are closed, and *downward deflections* of the traces correspond to channel openings. *B*, representative current amplitude histograms of single CFTR Cl^−^ channels in excised membrane patches from cells expressing wild-type (WT) CFTR, F508del-CFTR, and G551D-CFTR after filtering digitally at 50 Hz. The *continuous lines* represent the fit of Gaussian distributions to the data. The closed (*C*) and open (*O*) channel amplitudes are indicated; *S-CS*, a subconductance state of F508del-CFTR. For F508del-CFTR, a small leak current shifted the closed channel amplitude to ∼0.4 pA. (Current amplitude is calculated from the difference between the closed and open current amplitudes determined from the fit of Gaussian distributions to the data). *C* and *D*, summary single-channel current amplitude (*i*) and *P*_o_ or *P*_o(app)_ data. *Symbols*, individual values; *columns*, means ± S.E. (*error bars*) (wild type, *n* = 10; F508del-CFTR, *n* = 21; G551D-CFTR, *n* = 15); *, *p* < 0.05 *versus* wild-type CFTR; #, *p* < 0.05 *versus* F508del-CFTR fully open state; *ND*, not determined.

Fig. 1*A* shows representative single-channel recordings of wild-type CFTR, F508del-CFTR, and G551D-CFTR in excised inside-out membrane patches at 37 °C after channel activation was complete. The gating pattern of wild-type CFTR was characterized by bursts of channel openings interrupted by brief flickery closures, separated by longer closures between bursts (Fig. 1*A*). Consistent with previous data (*e.g.* see Refs. 29 and 31–33), F508del and G551D severely decreased the frequency of channel openings with the result that open probability (*P*_o_) (or apparent *P*_o_ (*P*_o(app)_) (30)) was reduced dramatically (Fig. 1, *A* and *D*). By contrast, neither F508del nor G551D affected current flow through fully open CFTR Cl^−^ channels (Fig. 1, *A–C*). However, close inspection of single-channel recordings revealed that F508del-CFTR also opened partially to a smaller current level, a subconductance state (Fig. 1*A*). These partial channel openings are more clearly seen in the single-channel current amplitude histogram of F508del-CFTR as a shoulder to the closed channel amplitude, 29% of the value of the fully open state (Fig. 1, *B* (*middle*) and *C*). Openings to subconductance states were observed in 37 of 39 recordings of F508del-CFTR in excised membrane patches and were always associated with channel deactivation (see below). By contrast, openings to subconductance states were only observed in 4 of 18 recordings of wild-type CFTR and were not associated with channel deactivation. We did not quantify the *P*_o_ of the subconductance state of F508del-CFTR because of its small current amplitude and variable gating pattern (76).

**FIGURE 2.**
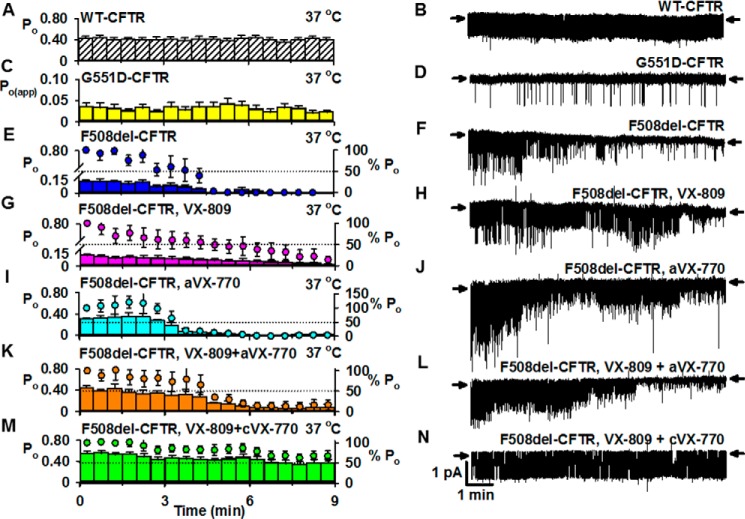
**Impact of ivacaftor and lumacaftor on the temporal instability of F508del-CFTR in excised inside-out membrane patches.** *A*, *C*, *E*, *G*, *I*, *K*, and *M*, time courses of *P*_o_; *B*, *D*, *F*, *H*, *J*, *L*, and *N*, representative recordings of wild-type CFTR, F508del-CFTR, and G551D-CFTR in excised inside-out membrane patches made in the continuous presence of ATP (1 mm) and PKA (75 nm) at 37 °C once channel activation was complete. For wild-type CFTR and G551D-CFTR, membrane patches were excised, and channels were activated and studied, all at 37 °C. For F508del-CFTR, membrane patches were excised, and channels were activated and potentiated at 27 °C to delay temperature-dependent channel deactivation. Only after F508del-CFTR was fully activated and potentiated was temperature increased to 37 °C and temporal stability evaluated. For F508del-CFTR, plasma membrane expression was rescued by incubation at 27 °C for 48–72 h (*E*, *F*, *I*, and *J*) or by treatment with lumacaftor (VX-809; 3 μm) at 37 °C for 24 h (*G*, *H*, *K*, *L*, *M*, and *N*). F508del-CFTR Cl^−^ channels were either acutely treated with ivacaftor (aVX-770; 10 μm) (*I–L*) or chronically incubated with ivacaftor (cVX-770; 1 μm) together with lumacaftor (VX-809; 3 μm) at 37 °C for 24 h (*M* and *N*). In *E*, *G*, *I*, *K*, and *M*, the *left* and *right ordinates* show *P*_o_ (*bars*) and normalized *P*_o_ (*circles*), respectively; *P*_o_ values were normalized to that measured immediately when temperature reached 37 °C (*t* = 0–30 s); *horizontal dotted lines* indicate 50% normalized *P*_o_. For wild-type and F508del-CFTR, *P*_o_ values were calculated for each 30-s interval, whereas for G551D-CFTR, *P*_o(app)_ values were calculated. Data are means ± S.E. (*error bars*) (wild-type CFTR, *n* = 8; G551D-CFTR, *n* = 8; F508del-CFTR, *n* = 10; F508del-CFTR, VX-809, *n* = 7; F508del-CFTR, aVX-770, *n* = 4; F508del-CFTR, VX-809 + aVX-770, *n* = 4; F508del-CFTR, VX-809 + cVX-770, *n* = 8). In *B*, *D*, *F*, *H*, *J*, *L*, and *N*, *arrows* denote the closed channel state, and *downward deflections* correspond to channel openings. In *A*, *E*, and *I*, some data were originally published in Wang *et al.* (32) (*A*, *n* = 4; *E*, *n* = 5; *I*, *n* = 4); other data are newly acquired.

The F508del mutation destabilizes full-length CFTR protein at the plasma membrane (14), leading to accelerated rundown of CFTR Cl^−^ channels at 37 °C (*e.g.* see Ref. 34). To investigate the impact of CF mutations on CFTR stability, we used *P*_o_ values to monitor the duration of channel activity in excised membrane patches at 37 °C (32). Fig. 2 illustrates the single-channel activity of wild-type CFTR, F508del-CFTR, and G551D-CFTR in excised membrane patches over the first 9 min of recordings at 37 °C after channel activation was complete (for details, see the legend to Fig. 2). Wild-type CFTR and G551D-CFTR both demonstrated stable and sustained channel activity, albeit that of G551D-CFTR was greatly reduced compared with wild-type CFTR (Fig. 2, *A–D*).

**FIGURE 3.**
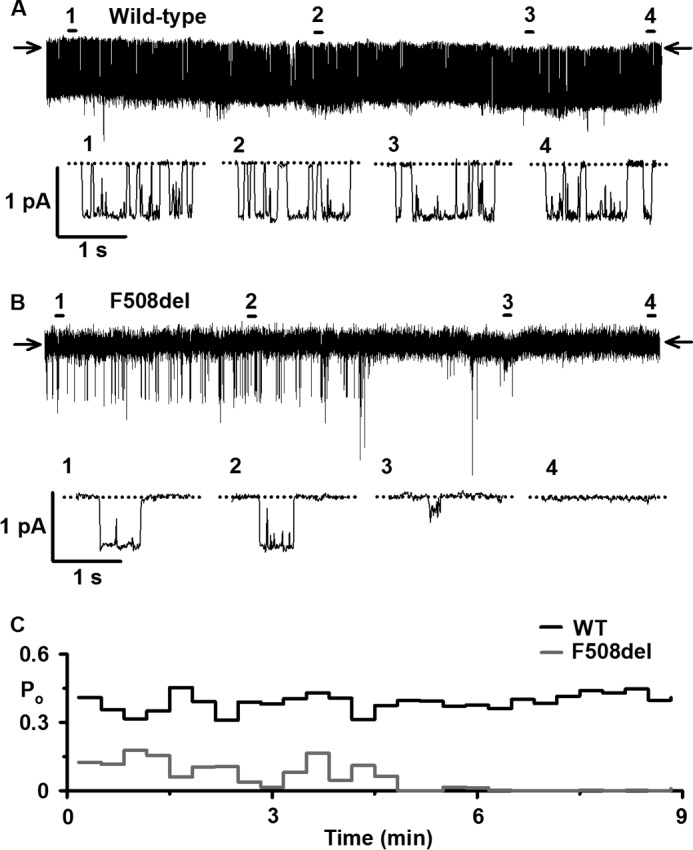
**Stability of wild-type and F508del-CFTR in excised inside-out membrane patches at 37 °C.** Shown are representative 9-min single-channel recordings (*A* and *B*) and corresponding *P*_o_ time courses (*C*) of wild-type and low temperature-rescued F508del-CFTR in excised inside-out membrane patches following full channel activation. ATP (1 mm) and PKA (75 nm) were continuously present in the intracellular solution; temperature was 37 °C. In *A* and *B*, four 2-s single-channel records labeled *1–4* indicated by *bars* are displayed on an expanded time scale *below* the 9-min recording to indicate channel activity at the beginning, in the middle, and at the end of the experiment. The *arrows* and *dotted lines* indicate where channels are closed, and *downward deflections* correspond to channel openings. In *C*, *P*_o_ values were calculated in 20-s intervals. For mean data, see Fig. 2, and for further information, see Figs. 1 and 2.

When rescued by low temperature incubation, F508del-CFTR exhibited sustained low level channel activity at 23 °C (32). By contrast, at 37 °C its activity was unstable and declined rapidly over time (Fig. 2, *E* and *F*). Comparison of normalized *P*_o_ values reveals that rundown of F508del-CFTR was 50% complete around 3 min and complete within 7 min (Fig. 2*E*). Visual inspection of single-channel records (Figs. 2*F* and 3) suggests that F508del-CFTR rundown involves both changes in channel gating and current flow through open channels. Reflecting the decline in F508del-CFTR *P*_o_, the change in gating pattern was characterized by reduced frequency of channel openings before complete channel deactivation (Figs. 2 (*E* and *F*) and 3). The change in current flow through F508del-CFTR accompanying channel rundown was characterized by increased frequency of partial channel openings to a subconductance state (*e.g.* Fig. 3). Thus, F508del causes CFTR instability by perturbing both channel gating and current flow through the CFTR pore.

##### Effects of Ivacaftor and Lumacaftor on the Instability of F508del-CFTR Cl^−^ Channels

Some studies suggest that ivacaftor destabilizes F508del-CFTR Cl^−^ channels (24, 25). To investigate the action of ivacaftor on F508del-CFTR, we studied single channels in excised membrane patches at 37 °C, comparing acute and chronic effects of the small molecule on F508del-CFTR Cl^−^ channels rescued by lumacaftor.

Fig. 2, *E–H*, compares the single-channel activity and stability of F508del-CFTR rescued by lumacaftor (3 μm for 24 h at 37 °C) with low temperature correction (48–72 h at 27 °C). Consistent with previous results (35), lumacaftor-rescued F508del-CFTR exhibited a gating defect, and its initial *P*_o_ at 37 °C following complete channel activation was not different from that of low temperature-rescued F508del-CFTR (*p* = 0.74; unpaired *t* test) (Fig. 2, *E* and *G*). Like low temperature-rescued F508del-CFTR, lumacaftor-rescued F508del-CFTR was unstable and deactivated over time (Fig. 2, *E–H*). Comparison of normalized *P*_o_ values suggests that lumacaftor-rescued F508del-CFTR Cl^−^ channels were slower to deactivate, but the difference was not statistically significant (low temperature-rescued F508del-CFTR, 50% normalized *P*_o_ = 3.5 min; lumacaftor-rescued F508del-CFTR, 50% normalized *P*_o_ = 5.0 min; *p* = 0.10; unpaired *t* test) (Fig. 2, *E* and *G*).

As we reported previously (32), when used acutely, ivacaftor (10 μm) initially potentiated markedly low temperature-rescued F508del-CFTR to achieve *P*_o_ values similar to those of wild-type CFTR in the absence of the small molecule (Fig. 2, *A* and *I*). However, ivacaftor-potentiated F508del-CFTR deactivated noticeably faster when compared with the absence of the drug (Fig. 2, *E* and *I*). Acute treatment of lumacaftor-rescued F508del-CFTR with ivacaftor (10 μm) also potentiated *P*_o_ markedly to attain wild-type levels of channel activity (Fig. 2, *A* and *K*). Comparison of normalized *P*_o_ values reveals that channel activities of ivacaftor-potentiated lumacaftor-rescued F508del-CFTR and ivacaftor-potentiated low temperature-rescued F508del-CFTR were similar (low temperature-rescued F508del-CFTR + acute ivacaftor, 50% normalized *P*_o_ = 3.5 min; lumacaftor-rescued F508del-CFTR + acute ivacaftor, 50% normalized *P*_o_ = 5.5 min; *p* = 0.05; unpaired *t* test) (Fig. 2, *I* and *K*). Nevertheless, *P*_o_ values declined noticeably faster in the presence of ivacaftor (Fig. 2, *G* and *K*).

In 8 of 12 experiments where F508del-CFTR Cl^−^ channels were rescued by chronic co-incubation with lumacaftor (3 μm) and ivacaftor (1 μm) for 24 h at 37 °C, we observed a strikingly different result (Fig. 2, *M* and *N*). First, the *P*_o_ of F508del-CFTR following complete channel activation was greatly elevated and similar to that of wild-type CFTR (*p* = 0.39; unpaired *t* test) (Fig. 2, *A* and *M*). Second, the temporal stability of these F508del-CFTR Cl^−^ channels was noticeably increased. Analysis of normalized *P*_o_ values indicates that 9 min after complete channel activation, F508del-CFTR rundown was markedly slowed and had not reached 50% complete (Fig. 2*M*). This result contrasts markedly with F508del-CFTR rescued by low temperature or lumacaftor, where F508del-CFTR rundown was complete or all but complete within 9 min (Fig. 2, *E* and *G*). It also contrasts with the destabilization of F508del-CFTR transepithelial Cl^−^ currents in CF airway epithelia chronically treated with lumacaftor and ivacaftor (24, 25). One possible explanation for this result is that in single-channel studies, we might have observed a minor population of F508del-CFTR Cl^−^ channels over a short time scale that does not represent the bulk behavior of the protein. Consistent with this idea, in 4 of 12 experiments, chronic co-incubation with lumacaftor and ivacaftor failed to rescue F508del-CFTR channel activity and stability. In these four experiments, F508del-CFTR Cl^−^ channels deactivated completely after activation at 27 °C, before temperature was increased to 37 °C for analysis of channel activity and temporal stability. We therefore decided to investigate bulk CFTR properties using purified proteins and membrane patches and polarized epithelia with large numbers of active channels that were followed over longer time periods.

##### Action of Ivacaftor and Lumacaftor on the Thermal Stability of CFTR Protein in Membranes and after Purification

To investigate the thermal stability of CFTR protein, we overexpressed wild-type CFTR, F508del-CFTR, and G551D-CFTR in yeast cells and purified CFTR-containing membranes (36, 37). To monitor the thermal stability of CFTR in membranes, we exploited a C-terminal GFP tag as a reporter. Detergent-solubilized CFTR was unfolded by incubation at different temperatures between 15 and 80 °C in 5 °C increments for 5 min at each temperature before analysis by SDS-PAGE. Thermal unfolding caused the appearance of SDS-resistant CFTR aggregates similar to those observed for CFTR (38) and other membrane proteins subject to equivalent treatment (39).

**FIGURE 4.**
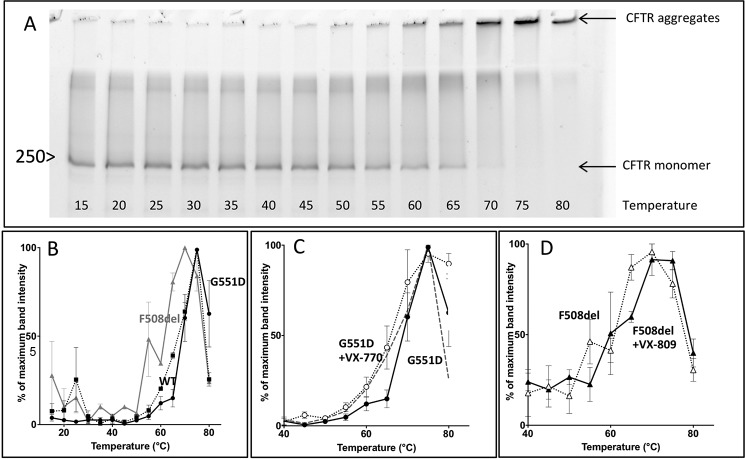
**Thermal stability of full-length CFTR protein in membranes and the effects of ivacaftor and lumacaftor.** *A*, representative gel showing the thermal stability of G551D-CFTR in solubilized microsomes probed by the formation of SDS-resistant aggregates (*top arrow*) and the concomitant disappearance of the monomeric CFTR band (*bottom arrow*). The position of the 250 kDa molecular mass marker is shown. CFTR was detected by the fluorescence of tagged GFP, which is stable up to 80 °C under the conditions used. *B–D*, summary data showing the thermal stability of wild-type CFTR, F508del-CFTR, and G551D-CFTR, the effects of ivacaftor (VX-770; 2 μm) on the thermal stability of G551D-CFTR and lumacaftor (VX-809; 2 μm) on the thermal stability of F508del-CFTR. Data are means ± S.E. (*error bars*) (*n* = 3).

Fig. 4*A* shows a representative SDS-polyacrylamide gel used to analyze the thermal stability of G551D-CFTR and Fig. 4, *B–D*, shows summary data for wild-type CFTR, F508del-CFTR, and G551D-CFTR. Fig. 4*B* demonstrates that wild-type and G551D-CFTR developed aggregation-detected unfolding curves with midpoints around 60 °C. In the initial stages of thermal denaturation, G551D-CFTR appeared slightly more stable than wild-type CFTR (Fig. 4*B*). By contrast, the unfolding transition of F508del-CFTR was broader and occurred at lower temperatures (Fig. 4*B*). Consistent with the functional stability of F508del-CFTR and G551D-CFTR (Fig. 2), these data argue that G551D-CFTR is stable, whereas F508del-CFTR is an unstable membrane protein.

To begin to investigate the action of lumacaftor on the thermal stability of F508del-CFTR protein, we incubated yeast cells expressing F508del-CFTR with lumacaftor (2 μm for 20 min at 4 °C). In contrast to functional studies, where acute and chronic effects of small molecules were evaluated, it was only feasible to test the acute effects of small molecules on CFTR protein produced in the yeast expression system. Fig. 4*D* demonstrates that lumacaftor induced some thermostabilization of F508del-CFTR, most noticeable around the midpoint of F508del-CFTR protein unfolding (control, 60.9 ± 1.8 °C; lumacaftor, 63.9 ± 2.8 °C; *n* = 3). By contrast, ivacaftor (2 μm for 20 min at 4 °C) was without effect on F508del-CFTR protein in this assay (*n* = 3) (77). Consistent with previous results (24), ivacaftor (2 μm for 20 min at 4 °C) induced a small, but significant, destabilization of G551D-CFTR (midpoint: control, 69.4 ± 2.9 °C; ivacaftor, 66.1 ± 1.1 °C; *n* = 3). Thus, ivacaftor restores to G551D-CFTR a thermal sensitivity similar to that of wild-type CFTR (Fig. 4*C*).

**FIGURE 5.**
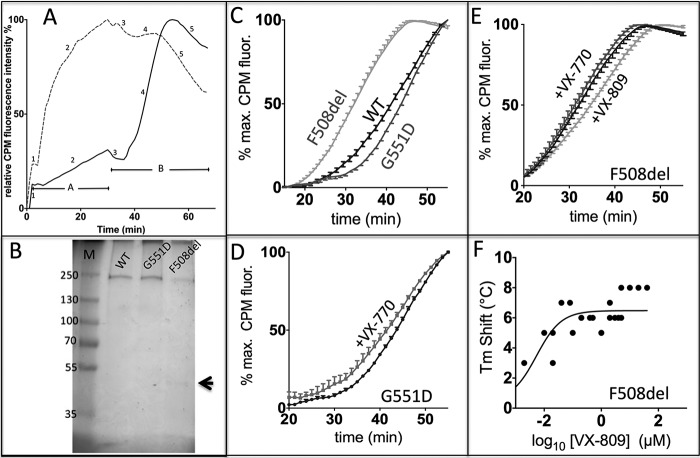
**F508del accelerates the thermal denaturation of purified CFTR protein.** *A*, unfolding of purified wild-type CFTR protein by thermal and chemical denaturation detected by changes in CPM fluorescence. The *continuous* and *dashed lines* show purified wild-type CFTR protein in DDM-containing buffer in the absence and presence of 4 m guanidine HCl. The experiment was initiated by injecting CPM dye into a cuvette containing purified wild-type CFTR protein at 10 °C and monitoring CPM fluorescence for 30 min (*period A*). At the end of period A, the purified wild-type CFTR protein was heated to 78 °C over a 30-min interval (*period B*). *Numbers* indicate changes in CPM fluorescence as follows: *1*, background fluorescence of CPM in buffer unbound to protein; *2*, kinetics of CPM binding to solvent-exposed cysteine residues; *3*, initial thermal quenching of CPM fluorescence as purified wild-type CFTR protein is heated; *4*, increase of CPM fluorescence as purified wild-type CFTR protein unfolds, exposing more cysteine residues to solvent; and *5*, continued thermal quenching of CPM fluorescence after complete unfolding of wild-type CFTR protein. *B*, silver-stained SDS-PAGE of purified protein samples for WT CFTR, F508del-CFTR, and G551D-CFTR. Wild-type CFTR and G551D-CFTR were purified by two chromatography steps, and F508del-CFTR was purified by one step. The *arrow* indicates the expected position of the main contaminating protein, ribosomal protein L3, which was removed by the second chromatography step. *C–E*, unfolding transitions of purified CFTR protein determined by changes in CPM fluorescence show the effects of F508del and G551D and the actions of ivacaftor (VX-770; 2 μm) and lumacaftor (VX-809; 2 μm). Samples of purified CFTR protein were heated at a rate of 1.2 °C/min. Data are means ± S.E. (*error bars*) (*n* = 3). *F*, concentration dependence of lumacaftor-induced stabilization of purified F508del-CFTR protein. *Filled circles*, data from individual experiments.

To better understand the impact of the F508del mutation on the thermal stability of CFTR protein, we applied a more sensitive stability assay using the cysteine-reactive fluorescent dye CPM (40) to purified CFTR protein solubilized from yeast cell membranes (Fig. 5, *A* and *B*). In aqueous buffers, CPM fluorescence is low, but it increases greatly upon formation of covalent adducts with cysteine residues in a protein (40). Fig. 5*A* demonstrates that CPM fluorescence increased noticeably when purified wild-type CFTR protein was added to a CPM-containing buffer as a result of adduct formation with surface-exposed cysteine residues. Complete denaturation of wild-type CFTR protein by guanidine HCl led to a 4-fold increase in CPM fluorescence over a period of about 30 min at 10 °C (Fig. 5*A*); half-time for cysteine adduct formation was about 7 min. Subsequent heating of the guanidine HCl-denatured wild-type CFTR protein did not increase further CPM fluorescence but rather led to a loss of fluorescence because of thermal quenching (Fig. 5*A*).

By contrast, in the absence of guanidine HCl, folded wild-type CFTR protein produced only a small increase in CPM fluorescence at 10 °C (Fig. 5*A*). Only with thermal denaturation of the purified wild-type CFTR protein by heating was a 3–4-fold increase in CPM fluorescence observed (Fig. 5*A*). After complete unfolding of purified wild-type CFTR protein at around 53 min, when the sample had reached ∼60 °C, a decrease in CPM fluorescence was observed due to thermal quenching. Because CPM fluorescence at any given time is determined by (i) the kinetics of protein unfolding, (ii) CPM-cysteine adduct formation, which is temperature-dependent, and (iii) the rate of heating, which leads to large hysteresis effects, this stability assay will produce different midpoint temperatures for protein unfolding, depending on these parameters. Hence, the midpoint temperature determined for the purified wild-type CFTR protein from the original data in Fig. 5*A* will be somewhat unreliable and, because of hysteresis, will probably overestimate the stability of purified wild-type CFTR protein. Hence, the assay was used for comparative purposes only under conditions where experimental parameters, such as the rate of heating, were constant (Fig. 5).

**FIGURE 6.**
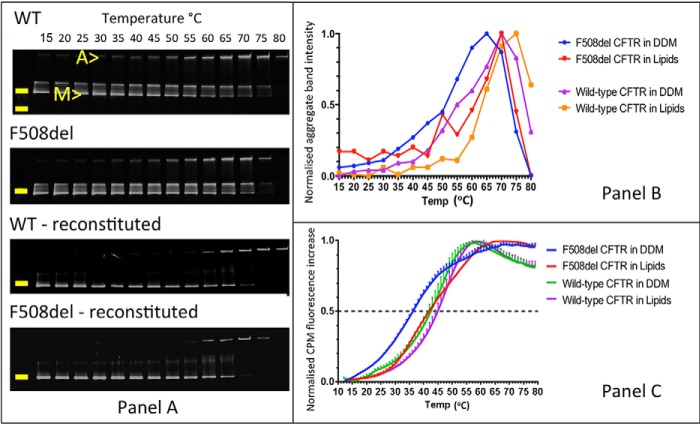
**Thermostability of purified WT CFTR and F508del-CFTR after reconstitution with lipid.** *A* and *B*, assessment of the stability of purified wild-type CFTR and F508del-CFTR using the thermal gel assay and detection of the GFP reporter tag fluorescence. An aggregate band *A* appears at higher temperatures with the concomitant disappearance of the monomer band *M* and an intermediate band. The positions of the 250 and 130 kDa molecular mass markers are indicated by the *yellow bars* for WT CFTR; in all other *panels*, only the 250 kDa marker is indicated. *C*, evaluation of the stability of purified wild-type and F508del-CFTR using CPM fluorescence. Data are means ± S.E. (*error bars*) (*n* = 3); for clarity, only one-half of the *error bars* are shown.

Fig. 5*C* demonstrates that purified F508del-CFTR protein displayed striking thermal destabilization compared with wild-type CFTR protein with a midpoint temperature shift of about 10 °C and a broader unfolding transition in the CPM assay. By contrast, purified G551D-CFTR protein exhibited cooperative unfolding with a stability comparable with or even greater than that of purified wild-type CFTR protein (Fig. 5*C*). Interestingly, the overall comparative stability of the purified CFTR protein was not noticeably different from that of CFTR protein in membranes assayed by SDS-PAGE (Figs. 4 and 5*C*). Hence, there was no evidence for effects of the protein purification process *per se* on CFTR stability. Nevertheless, the destabilizing effects of detergent solubilization and the stabilizing effects of a lipid bilayer on membrane proteins have been described frequently (41). For CFTR, we observed similar behavior (37), with thermal stabilization of wild-type CFTR and F508del-CFTR in the presence of a lipid bilayer (Fig. 6). Of note, the difference in stability between wild-type CFTR and F508del-CFTR is less in a lipid bilayer, suggesting that detergent might exacerbate the instability of F508del-CFTR protein.

Fig. 5*E* demonstrates that treatment of purified F508del-CFTR protein with lumacaftor (2 μm for 20 min at 4 °C) partially recovered protein thermostability similar to the drug's effects on F508del-CFTR in membranes (Fig. 4*D*). However, analysis of the concentration dependence of this effect indicated that maximal recovery of F508del-CFTR protein stability was achieved at about 2 μm lumacaftor (Fig. 5*F*). There was no narrowing of the unfolding transition after the addition of lumacaftor (Fig. 5*E*). Interestingly, the addition of nucleotide (2 mm ATP) restored a similar amount of stability to purified F508del-CFTR protein as lumacaftor (2 μm), and the effects were not synergistic (*n* = 3) (77). In contrast to the effects of lumacaftor, ivacaftor (2 μm for 20 min at 4 °C) caused a small, but significant, thermo-destabilization of purified F508del-CFTR protein (Fig. 5*E*). Consistent with previous results (24) and Fig. 4*C*, ivacaftor (2 μm for 20 min at 4 °C) also caused a small, but significant, destabilization of purified G551D-CFTR protein, restoring wild-type levels of protein stability to the mutant protein (Fig. 5*D*). Thus, F508del exerts similar effects on purified CFTR protein and single CFTR Cl^−^ channels, whereas lumacaftor restores some stability to both pure protein and single CFTR Cl^−^ channels, but ivacaftor destabilizes them. Interestingly, the combination of both drugs added acutely neither stabilized nor destabilized the purified protein. It is possible that the drugs mutually cancel out. However, lumacaftor must be given chronically to cells to rescue F508del-CFTR. For reasons of cost, it was not possible to grow yeast cells in the presence of lumacaftor in sufficient numbers to purify F508del-CFTR.

##### F508del-CFTR Exhibits Two Populations of Cl^−^ Channels after Chronic Treatment with Lumacaftor and Ivacaftor

**FIGURE 7.**
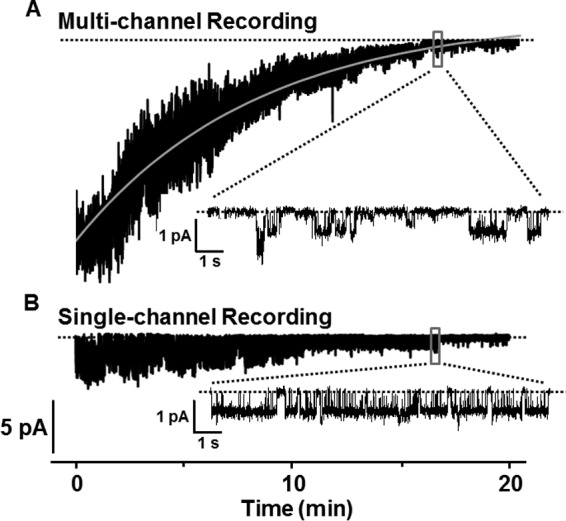
**Instability of F508del-CFTR Cl^−^ currents in excised inside-out membrane patches.** *A* and *B*, representative long duration recordings of F508del-CFTR Cl^−^ currents and channels in excised inside-out membrane patches following F508del-CFTR rescue by treatment of cells with lumacaftor (3 μm) and ivacaftor (1 μm) for 24 h at 37 °C. F508del-CFTR Cl^−^ channels were activated fully with ATP (1 mm) and PKA (75 nm) at 27 °C before the temperature was increased to 37 °C and the recordings commenced. ATP (1 mm) and PKA (75 nm) were continuously present in the intracellular solution for the duration of the recordings. Beneath each 20-min recording, the indicated 10-s period is shown on an expanded time scale. The *dotted lines* indicate where channels are closed, and *downward deflections* of the *traces* correspond to channel openings. In *A*, the *continuous gray line* shows the fit of a single exponential function to determine the time constant for the deactivation of F508del-CFTR Cl^−^ currents. Similar results were observed in other experiments (F508del-CFTR Cl^−^ currents, *n* = 3; F508del-CFTR Cl^−^ channels, *n* = 5).

To investigate the effects of lumacaftor and ivacaftor on a population of F508del-CFTR Cl^−^ channels, we studied large numbers of active channels in excised membrane patches and transepithelial Cl^−^ currents in polarized epithelia. Fig. 7*A* shows the time course of F508del-CFTR Cl^−^ currents in an excised inside-out membrane patch containing multiple active F508del-CFTR Cl^−^ channels; similar results were observed in three other experiments. The plasma membrane expression of these F508del-CFTR Cl^−^ channels was rescued by chronic incubation of baby hamster kidney (BHK) cells expressing F508del-CFTR with lumacaftor (3 μm) and ivacaftor (1 μm) for 24 h at 37 °C. Immediately following CFTR activation with PKA (75 nm) and ATP (1 mm) at 37 °C, large numbers of F508del-CFTR Cl^−^ channels were observed (Fig. 7*A*). Despite the continuous presence of ATP and PKA in the intracellular solution, over time there was a progressive loss of F508del-CFTR channel activity with a time constant of 405 s (Fig. 7*A*). After about 15 min, only two F508del-CFTR Cl^−^ channels remained. Their behavior, including gating pattern and partial openings to a subconductance state, resembled that of low temperature-rescued F508del-CFTR Cl^−^ channels that had undergone channel rundown at 37 °C (Figs. 3 and 7*A*). As we reported previously (32), once F508del-CFTR Cl^−^ channels had undergone rundown, they could not be reactivated with fresh ATP and PKA.

In six excised inside-out membrane patches with small numbers of F508del-CFTR Cl^−^ channels studied over a prolonged period, F508del-CFTR rescued by chronic treatment with lumacaftor and ivacaftor exhibited a different pattern of behavior. Fig. 7*B* shows one such example. In this recording, ATP (1 mm) and PKA (75 nm) activated a small number of F508del-CFTR Cl^−^ channels, but these F508del-CFTR Cl^−^ channels possessed high activity and greater temporal stability with the result that after 15 min, the remaining F508del-CFTR Cl^−^ channel had a gating pattern characterized by frequent prolonged bursts of channel openings (Fig. 7*B*). The high activity and notable stability of these F508del-CFTR Cl^−^ channels are reminiscent of those observed in Fig. 2 (*M* and *N*), following chronic treatment with lumacaftor (3 μm) and ivacaftor (1 μm) for 24 h at 37 °C. We interpret these data to suggest that chronic treatment with lumacaftor and ivacaftor restores high levels of channel activity and prolonged stability to a small subpopulation of F508del-CFTR Cl^−^ channels. However, the majority of rescued F508del-CFTR Cl^−^ channels are relatively unstable at 37 °C.

To explore this hypothesis, we studied CFTR-mediated transepithelial Cl^−^ currents in Fischer rat thyroid (FRT) epithelia expressing F508del-CFTR. We rescued the apical membrane expression of F508del-CFTR by treating FRT epithelia with lumacaftor (3 μm) for 24 h at 37 °C and either acutely or chronically exposed these FRT epithelia to ivacaftor (1 μm). As controls, we studied FRT epithelia expressing wild-type CFTR and G551D-CFTR. To identify cAMP-activated Cl^−^ currents carried by CFTR, we used the thiazolidinone CFTR inhibitor CFTR_inh_-172 (42). To specifically investigate the bulk population of CFTR Cl^−^ channels present at the apical membrane, FRT epithelia were treated with the protein synthesis inhibitor cycloheximide (50 μg/ml) 15 min before mounting FRT epithelia in Ussing chambers and recording CFTR-mediated transepithelial Cl^−^ currents at *t* = 0 h. The remaining FRT epithelia were incubated with cycloheximide (50 μg/ml) for 2, 4, or 6 h before use. Cycloheximide treatment was without effect on epithelial integrity (as measured by transepithelial resistance; *R*_t_) (*e.g.* for wild-type CFTR-expressing FRT epithelia chronically treated with ivacaftor: at 0 h, *R*_t_ = 1.79 ± 0.32 kiloohms cm^2^; at 6 h, *R*_t_ = 3.33 ± 0.96 kiloohms cm^2^; *n* = 6).

**FIGURE 8.**
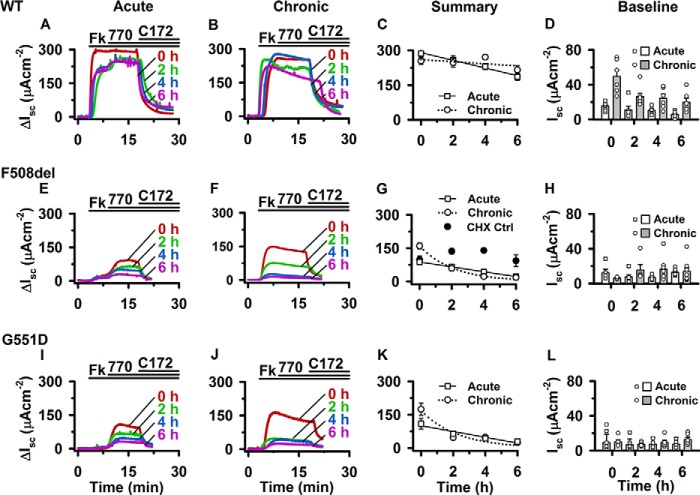
**Deactivation of CFTR-mediated transepithelial Cl^−^ currents after acute or chronic ivacaftor treatment.** Shown are representative Ussing chamber recordings of WT CFTR (*A* and *B*), lumacaftor-rescued (VX-809; 3 μm for 24 h at 37 °C) F508del-CFTR (*E* and *F*), and G551D-CFTR (*I* and *J*). FRT epithelia were incubated for 24 h at 37 °C in the absence (*A*, *E*, and *I*; *Acute*) or presence (*B*, *F*,and *J*; *Chronic*) of ivacaftor (VX-770; 1 μm); F508del-CFTR-expressing FRT epithelia were co-incubated with lumacaftor and ivacaftor. Fifteen minutes before *t* = 0 h, FRT epithelia were treated with cycloheximide (50 μg/ml), added to both the apical and basolateral solutions. At the indicated times, FRT epithelia were mounted in Ussing chambers, and CFTR Cl^−^ currents were activated with forskolin (*Fk*; 10 μm), potentiated with ivacaftor (VX-770 (*770*); 1 μm), and inhibited by CFTR_inh_-172 (*C172*; 10 μm); *continuous lines* indicate the presence of different compounds in the apical solution; cycloheximide (50 μg/ml) was present in the apical and basolateral solutions during *I*_sc_ recordings. Data are normalized to baseline current so that Δ*I*_sc_ represents the change in transepithelial current after CFTR activation by forskolin. *C*, *G*, and *K* (*Summary*), magnitude of ivacaftor-potentiated Δ*I*_sc_ for WT CFTR, lumacaftor-rescued F508del-CFTR, and G551D-CFTR at different times after cycloheximide (50 μg/ml) treatment. Data are means ± S.E. (*error bars*) (*n* = 5–10); the *continuous* and *dotted lines* show the fits of linear and single exponential functions, respectively, to WT CFTR, F508del-CFTR, and G551D-CFTR Cl^−^ currents acutely and chronically treated with ivacaftor. *G*, as a control, the magnitude of lumacaftor-rescued F508del-CFTR Cl^−^ current chronically treated with ivacaftor (1 μm for 24 h at 37 °C) but untreated with cycloheximide is shown (0.1% (v/v) DMSO; *CHX Ctrl*). *D*, *H*, and *L* (*Baseline*), magnitude of absolute *I*_sc_ before CFTR activation for WT CFTR-, lumacaftor-rescued F508del-CFTR-, and G551D-CFTR-expressing FRT epithelia acutely or chronically treated with ivacaftor. *Symbols*, individual values; *columns*, means ± S.E. (*n* = 5–10).

In wild-type CFTR expressing FRT epithelia, forskolin (10 μm) activated rapidly large CFTR-mediated Cl^−^ currents of similar magnitude in epithelia acutely or chronically treated with ivacaftor (1 μm) (Fig. 8, *A* and *B*). The decline of CFTR-mediated Cl^−^ current after chronic ivacaftor treatment (Fig. 8*B*) might be a consequence of treating FRT chronically with a drug (43), CFTR inhibition by high concentrations of potentiators (44), or the dephosphorylation of CFTR (for discussion, see Ref. 45). Consistent with previous results (24, 25), when wild-type CFTR-expressing FRT epithelia were chronically treated with ivacaftor (1 μm), CFTR Cl^−^ currents were not potentiated when ivacaftor (1 μm) was subsequently added acutely (Fig. 8*B*). Fig. 8*C* demonstrates that there was a slow, steady decline in the magnitude of ivacaftor-potentiated CFTR-mediated Cl^−^ currents over 6 h, and the rate of decline was unaffected by pretreatment with ivacaftor (1 μm) (*p* = 0.084). However, chronic ivacaftor treatment enhanced the magnitude of baseline *I*_sc_ 2.8-fold in FRT epithelia expressing wild-type CFTR (Fig. 8*D*). For two reasons, this enhanced baseline *I*_sc_ was CFTR-dependent; first, its inhibition by CFTR_inh_-172 (control, *I*_sc_ = 41 ± 9 μA/cm^2^; CFTR_inh_-172 (10 μm), *I*_sc_ = 20 ± 3 μA/cm^2^; *n* = 6) and, second, the attenuation of baseline *I*_sc_ in FRT epithelia expressing lumacaftor-rescued F508del-CFTR and G551D-CFTR (Fig. 8, *H* and *L*).

Fig. 8*E* demonstrates that forskolin (10 μm) activated small CFTR-mediated transepithelial Cl^−^ currents in lumacaftor-rescued F508del-CFTR FRT epithelia that were potentiated by acute treatment with ivacaftor (1 μm). However, over a 6-h period in the continuous presence of cycloheximide (50 μg/ml), the magnitude of the ivacaftor-potentiated CFTR-mediated Cl^−^ current declined 3.9-fold (Fig. 8, *E* and *G*). By contrast, when F508del-CFTR-expressing FRT epithelia were chronically treated with lumacaftor (3 μm) and ivacaftor (1 μm) for 24 h at 37 °C, the magnitude of the CFTR-mediated Cl^−^ current activated by forskolin (10 μm) was initially increased markedly, and at all time points the current was unaffected by acute treatment with ivacaftor (1 μm) (Fig. 8*F*). Fig. 8*G* demonstrates that in cycloheximide-treated lumacaftor-rescued F508del-CFTR FRT epithelia, CFTR-mediated Cl^−^ currents were initially enhanced 1.8-fold compared with acute treatment with ivacaftor (1 μm). However, these enhanced CFTR-mediated Cl^−^ currents in chronically treated F508del-CFTR epithelia decayed significantly faster than those observed in F508del-CFTR epithelia acutely treated with ivacaftor (1 μm) (*p* < 0.001) (Fig. 8*G*). As a control, we omitted cycloheximide (50 μg/ml) treatment. Fig. 8*G* demonstrates that in the absence of cycloheximide, the magnitude of CFTR-mediated Cl^−^ current was sustained. Interestingly, Fig. 8 (*I–K*) demonstrates that similar results were observed when G551D-CFTR-expressing FRT epithelia were chronically treated with ivacaftor (1 μm). The magnitude of CFTR-mediated Cl^−^ current was initially enhanced 1.6-fold, but it decayed significantly faster than that of G551D-CFTR FRT epithelia acutely treated with ivacaftor (1 μm) (*p* = 0.036) (Fig. 8*K*). These data are consistent with previous results demonstrating that chronic exposure of F508del-CFTR-expressing epithelia to ivacaftor destabilizes CFTR-mediated Cl^−^ currents (24, 25). Thus, our data suggest that chronic treatment of cells expressing recombinant F508del-CFTR with ivacaftor restored high levels of channel activity and prolonged stability only to a small subpopulation of F508del-CFTR Cl^−^ channels.

## Discussion

This study investigated the instability of F508del-CFTR and its rescue by lumacaftor and ivacaftor. We demonstrated that F508del destabilized purified full-length CFTR protein by about 10 °C and caused time-dependent deactivation of individual CFTR Cl^−^ channels by altering channel gating and current flow through the CFTR pore. Lumacaftor and ivacaftor had complex effects on F508del-CFTR. Interpretation of the data suggests that combination therapy with lumacaftor and ivacaftor rescues a small subpopulation of F508del-CFTR Cl^−^ channels.

Previous studies demonstrate that the F508del mutation causes thermoinstability of isolated human NBD1 (*e.g.* see Refs. 46–48). Biophysical studies of NBD1 thermal and chemical denaturation identified two sequential steps in NBD1 unfolding and revealed that F508del targets the first step, leading to a partially unfolded, aggregation-prone structure (47, 49). Comparison of the present results with previous studies suggests that F508del has greater impact on the thermostability of full-length CFTR protein than isolated human NBD1 (NBD1, 6–8 °C (47, 48); full-length CFTR, ∼10 °C (present study)). One potential explanation for this difference is the experimental conditions employed to investigate the thermostability of full-length CFTR protein. Nevertheless, the greater thermosensitivity of full-length F508del-CFTR protein is consistent with the disruption of domain-domain interactions critical for correct CFTR assembly and function following the loss of Phe-508 (8–11, 48, 50).

Single-channel studies using excised membrane patches and planar lipid bilayers highlight the consequences of F508del-CFTR instability for channel function. At 37 °C, F508del-CFTR Cl^−^ channels deactivate promptly, leading to an irreversible loss of channel activity in the continuous presence of PKA and ATP (*e.g.* see Ref. 34) (present study). Consistent with the data of Aleksandrov *et al.* (34), but not that of Wang *et al.* (51) and Liu *et al.* (52), we found that the deactivation of F508del-CFTR Cl^−^ channels involved both changes in channel gating and current flow through open channels. Moreover, persistence of F508del-CFTR at the plasma membrane at 37 °C is considerably longer (*t*_½_ ≤ 4 h) (14) than its loss of function in cell-free membrane patches (<5 min) (present study), concurring with the conclusion that F508del-CFTR has inherent instability when activated (52). Interestingly, several studies have identified revertant (second site) mutations in *cis* with F508del, including missense mutations and deletions, which abrogate the deactivation of F508del-CFTR Cl^−^ channels (34, 51–53). Taken together, these data argue that F508del-CFTR thermoinstability is a consequence of changes in CFTR structure, not loss of F508del-CFTR tethering to CFTR-interacting proteins (54).

F508del is located at a critical interface in the CFTR gating pathway, which transduces conformation changes in the NBDs to the membrane-spanning domains (MSDs) to control gating of the channel pore (9, 10). Thus, changes in channel gating during F508del-CFTR deactivation might be a consequence of disabled communication between the NBDs and MSDs. However, the literature suggests two alternative explanations: first, destabilization of the full and partial NBD1-NBD2 dimer configurations (55) in the ATP-driven NBD dimerization model of channel gating (4, 56) and, second, defective regulation of F508del-CFTR Cl^−^ channels by PKA-dependent phosphorylation of the R domain (57). Disabled communication between the NBDs and MSDs also provides a potential explanation for instability of the CFTR pore during F508del-CFTR deactivation. Cui *et al.* (58) identified salt bridges between transmembrane segments in the MSDs that stabilize the open channel configuration. We speculate that defective signaling from the NBDs during F508del-CFTR deactivation might destabilize these salt bridges, favoring opening of the CFTR pore to a subconductance state. Alternatively, defective signaling from the NBDs might prevent full opening of a gate located in the pore constriction (59). Future studies should explore these possibilities.

Lumacaftor and ivacaftor are the first drugs for CF designed to directly target defects in CFTR. Lumacaftor acts cotranslationally to allow some F508del-CFTR to escape intracellular degradation and traffic to the plasma membrane (16). It also acts posttranslationally to stabilize F508del-CFTR at the plasma membrane (60). Consistent with this latter result, the present results demonstrate that once at the plasma membrane, lumacaftor-rescued F508del-CFTR is more stable than low temperature-rescued F508del-CFTR. The data argue that lumacaftor binds directly to the mutated protein and stabilizes it to some extent. This interpretation is further supported by our experiments performed on single Cl^−^ channels and purified protein (Figs. 2–6) as well as previously published experiments with N-terminal fragments of CFTR (61, 62). When lumacaftor was added acutely to purified F508del-CFTR protein, its thermostabilization effect saturated at around 2 μm, which corresponds closely to the functional effects of the drug added chronically (16). This direct correlation between thermal stabilization and functional rescue of F508del-CFTR provides some evidence that the principal defect of the F508del mutation might be reduced thermostability, consistent with the results of biophysical studies of NBD1 thermal and chemical denaturation (47, 49).

Ivacaftor robustly potentiates CFTR channel gating to restore Cl^−^ channel function to CF mutants (17, 63). In contrast to other CFTR potentiators that enhance ATP-dependent channel gating at the NBDs (33, 64), the action of ivacaftor is ATP-independent (65, 66). Using computer modeling, Cholon *et al.* (24) demonstrate that ivacaftor destabilizes the structure of CFTR. Whereas this destabilization is beneficial to the rigid structure of G551D-CFTR, it is deleterious to the delicate structure of F508del-CFTR (24), providing an explanation for the drug's effects on purified CFTR protein and single channels observed in the present study.

Our data raise the interesting possibility that chronic co-incubation of cells expressing F508del-CFTR with lumacaftor and ivacaftor rescues a small subpopulation of F508del-CFTR Cl^−^ channels with distinct characteristics. Consistent with previous results (35), the single-channel activity of low temperature- and lumacaftor-rescued F508del-CFTR Cl^−^ channels was closely comparable. By contrast, F508del-CFTR Cl^−^ channels rescued by chronic co-incubation with lumacaftor and ivacaftor were characterized by greatly increased activity and temporal stability. At the present time, we do not know the identity of this subpopulation of F508del-CFTR Cl^−^ channels, nor whether both populations are found in one cell, although this is likely (67). The simplest interpretation of the data is that chronic co-incubation with lumacaftor and ivacaftor directly targets F508del-CFTR to rescue protein folding defects and improve channel gating and stability. Consistent with this idea, studies docking lumacaftor with CFTR homology models argue that it binds directly to CFTR at the NBD1-MSD1/2 interface (22, 23, 68). Although the binding site for ivacaftor on CFTR is currently unknown, single-channel studies of purified recombinant protein (65) argue that CFTR, itself, is targeted by ivacaftor, whereas studies of wild-type CFTR and CF mutants argue that amino acid sequences within the MSDs probably form its binding site (63, 66). Thus, chronic co-incubation would favor the interaction of lumacaftor and ivacaftor with F508del-CFTR, potentially leading to improved correction of a subset of F508del-CFTR Cl^−^ channels.

Alternatively, chronic co-incubation might rescue a subpopulation of F508del-CFTR Cl^−^ channels by affecting membrane lipids. In support of this idea, Baroni *et al.* (69) demonstrated that ivacaftor accumulates in the inner leaflet of lipid bilayers. Moreover, Abu-Arish *et al.* (67) identified two populations of CFTR Cl^−^ channels at the plasma membrane distinguished by their interaction with cholesterol-rich membrane microdomains. Whereas the majority of CFTR Cl^−^ channels at the plasma membrane were mobile, some demonstrated cholesterol-dependent confinement (67). Future studies should investigate further how membrane lipids modulate CFTR expression, stability, and function and its interaction with small molecules.

In conclusion, the F508del mutation causes a large destabilization of full-length CFTR protein, noticeably greater than that reported previously for isolated NBD1 (46–48). Consistent with these data, the time-dependent deactivation of F508del-CFTR Cl^−^ channels in excised membrane patches at 37 °C involves changes in both channel gating and Cl^−^ conductance. Together, the data argue that the F508del mutation has wide ranging impact on CFTR structure. The clinically approved CFTR modulators lumacaftor and ivacaftor had complex effects on CFTR stability. Of special note, chronic co-incubation of cells expressing F508del-CFTR with lumacaftor and ivacaftor greatly increased the activity and stability of a small subpopulation of F508del-CFTR Cl^−^ channels. The challenge for the future is to identify strategies to convert the majority of unstable F508del-CFTR Cl^−^ channels into this restored form.

## Experimental Procedures

### 

#### 

##### Cells and Cell Culture

We used BHK cells stably expressing wild-type and F508del human CFTR and FRT epithelial cells stably expressing F508del and G551D human CFTR (70, 71). Cells were generous gifts from M. D. Amaral (University of Lisboa; BHK cells) and L. J. V. Galietta (Instituto Giannina Gaslini; FRT cells). Unless otherwise indicated, cells were cultured and used as described previously (43, 72). When testing the effects of chronic drug treatments on F508del-CFTR-expressing BHK cells (but not F508del-CFTR-expressing FRT epithelia), the fetal bovine serum concentration of media was reduced to 1%. The single-channel behavior of wild-type human CFTR in excised membrane patches from different mammalian cell lines is equivalent (73).

##### Patch Clamp Experiments

CFTR Cl^−^ channels were recorded in excised inside-out membrane patches using an Axopatch 200A patch clamp amplifier and pCLAMP software (both from Molecular Devices, Sunnyvale, CA) (74). The pipette (extracellular) solution contained 140 mm *N*-methyl-d-glucamine, 140 mm aspartic acid, 5 mm CaCl_2_, 2 mm MgSO_4_, and 10 mm TES, adjusted to pH 7.3 with Tris ([Cl^−^], 10 mm). The bath (intracellular) solution contained 140 mm *N*-methyl-d-glucamine, 3 mm MgCl_2_, 1 mm CsEGTA, and 10 mm TES, adjusted to pH 7.3 with HCl ([Cl^−^], 147 mm; free [Ca^2+^] < 10^−8^ m) and was maintained at 27 or 37 °C using a temperature-controlled microscope stage (Brook Industries, Lake Villa, IL).

After excision of inside-out membrane patches, we added the catalytic subunit of PKA (75 nm) and ATP (1 mm) to the intracellular solution within 2 min of patch excision to activate CFTR Cl^−^ channels. To minimize channel rundown, we added PKA to all intracellular solutions and maintained the ATP concentration at 1 mm and clamped voltage at −50 mV. To investigate the temporal stability of wild-type CFTR and G551D-CFTR, membrane patches were excised, channels were activated, and their stability was assessed all at 37 °C. However, to study the instability of F508del-CFTR, membrane patches were excised and activated at 27 °C to delay temperature-dependent channel deactivation. In some experiments, F508del-CFTR Cl^−^ channels were potentiated by the addition of ivacaftor (10 μm) to the intracellular solution in the continuous presence of ATP (1 mm) and PKA (75 nm). Once F508del-CFTR Cl^−^ channels were fully activated and potentiated when using ivacaftor, the temperature of the intracellular solution was increased to 37 °C, which took 2–3 min. To monitor CFTR temporal stability, we calculated *P*_o_ and normalized *P*_o_ in 30-s intervals over a 9-min period (32). To investigate the temporal stability of F508del-CFTR Cl^−^ currents, we studied membrane patches containing multiple active channels over a 20-min period.

For single-channel studies, we used membrane patches containing ≤5 active channels (wild-type CFTR, number of active channels (*n*) ≤ 4; F508del-CFTR, *n* ≤ 5). To determine channel number, we used the maximum number of simultaneous channel openings observed during the course of an experiment, as described previously (33). Because of the extremely low activity of G551D-CFTR (29, 30), we did not determine channel number for G551D-CFTR. Instead, we express G551D-CFTR channel activity as *P*_o(app)_ (30).

We recorded, filtered and digitized data as described previously (74). To measure single-channel current amplitude, Gaussian distributions were fitted to current amplitude histograms. To measure *P*_o_, lists of open and closed times were created using a half-amplitude crossing criterion for event detection, and dwell time histograms were constructed and fitted as described previously (74). For the purpose of illustration, single-channel records were filtered at 500 Hz and digitized at 5 kHz before file size compression (Fig. 1, 5-fold data reduction; Figs. 2, 3, and 7, 50-fold).

##### Ussing Chamber Studies

CFTR-mediated transepithelial Cl^−^ currents in FRT epithelia were recorded using a large Cl^−^ concentration gradient to magnify current size without permeabilizing the basolateral membrane (72). FRT epithelia were mounted in Ussing chambers (Warner Instrument Corp., Dual Channel Chamber; Harvard Apparatus Ltd., Edenbridge, UK). The solution bathing the basolateral membrane contained 140 mm NaCl, 5 mm KCl, 0.36 mm K_2_HPO_4_, 0.44 mm KH_2_PO_4_, 1.3 mm CaCl_2_, 0.5 mm MgCl_2_, 10 mm HEPES, and 4.2 mm NaHCO_3_, adjusted to pH 7.2 with Tris ([Cl^−^], 149 mm). The solution bathing the apical membrane was identical to that of the basolateral solution with the exception that 133.3 mm sodium gluconate + 2.5 mm NaCl and 5 mm potassium gluconate replaced 140 mm NaCl and 5 mm KCl, respectively, to create a transepithelial Cl^−^ concentration gradient ([Cl^−^], 14.8 mm). To compensate for calcium buffering by gluconate, we used 5.7 mm Ca^2+^ in the apical solution. All solutions were maintained at 37 °C and bubbled continuously with 5% CO_2_.

After cancelling voltage offsets, we clamped transepithelial voltage (referenced to the basolateral solution) at 0 mV and recorded *I*_sc_ continuously using an epithelial voltage clamp amplifier (Warner Instrument Corp., model EC-825; Harvard Apparatus Ltd.), digitizing data as described previously (75). The resistance of the filter and solutions, in the absence of cells, was subtracted from all measurements. Under the experimental conditions that we used, flow of current from the basolateral to the apical solution corresponds to Cl^−^ movement through open CFTR Cl^−^ channels and is shown as an upward deflection. For the purpose of illustration, *I*_sc_ time courses are displayed as Δ*I*_sc_ with the *I*_sc_ value immediately preceding forskolin addition designated as 0 μA/cm^2^; file sizes were compressed by 100-fold data reduction.

##### Thermal Gel Analysis

Microsomes from *Saccharomyces cerevisiae* expressing GFP-tagged CFTR (36, 37) were solubilized with 2% (w/v) dodecyl-β-d-maltoside (DDM) for 2 h and then centrifuged at 100,000 × *g* for 1 h at 4 °C. DDM-solubilized material in the supernatant was collected and diluted 1:4 with CFTR buffer (50 mm Tris, pH 8.0, 10% (v/v) glycerol) to a final concentration of 0.5% (w/v) DDM. The sample was then placed into an Eppendorf tube in a water bath, and the temperature was increased from 15 to 80 °C at 2.5 °C/min between sampling temperatures. Every 5 °C, the sample was incubated for 5 min before a 20-μl aliquot was taken and quenched immediately on ice for 10 min before the addition of 20 μl of SDS-loading buffer. All of the samples collected at different temperatures were analyzed on the same SDS-polyacrylamide gel, and CFTR-containing bands were quantified using the GFP fluorescence signal and ImageJ software.

##### Protein Purification

CFTR protein was initially purified as described by O'Ryan *et al.* (37) using DDM. The nickel-nitrilotriacetic acid-purified protein (first step) was then loaded onto a pre-equilibrated 1-ml FLAG affinity column (Sigma) and washed with 10 column volumes of buffer before bound protein was eluted with 100 μg/ml FLAG peptide (Sigma) in the same DDM-containing buffer. The protein yield was about 20% of the protein initially added to the FLAG affinity column. For the F508del-CFTR protein, which had a lower level of expression, the FLAG purification step was omitted. The purity of the final fractions was assessed by SDS-PAGE (see Fig. 5*B*). Purified CFTR protein was reconstituted with lipids as described (37).

##### CPM Binding Analysis

This fluorescence assay (40) is sensitive to plasticizers that leach out of plastic. Hence, buffer components, detergent-containing buffers, and stocks of the fluorochrome *N*-[4-(7-diethylamino-4-methyl-3-coumarinyl)phenyl]maleimide (CPM) were stored in glass containers; CPM was stored in the dark at −20 °C. Immediately before use, CPM stock solutions were diluted into buffer using a glass microsyringe. Dithiothreitol (DTT) was removed from buffers and protein-containing samples before the addition of CPM. CPM had a measurable background fluorescence due to detergent micelles (77). Hence, the buffer control signal was subtracted from test signals. Protein at 5–7 μg/ml (about 25 nm) was added to buffer containing CPM (0.7 μg/ml, 2.4 μm) and DDM (0.1% w/v) (37). When used at ≤10 μg/ml (34 μm), CPM concentration was without effect on the measured unfolding transition of CFTR. However, at higher CPM concentrations, the measured unfolding transition of CFTR shifted to higher temperatures. After incubation at 10 °C for 20 min, a temperature ramp of 1.5–2.5 °C/min was initiated. At faster heating rates, hysteresis or lag was apparent, at least partly due to the finite time required for CPM binding to occur. CPM fluorescence was recorded using a Cary Eclipse fluorimeter with excitation at 387 nm (5-nm slit) and emission at 463 nm (5-nm slit) in a 150 μl, 1-cm path length quartz fluorescence cuvette (Hellma).

##### Materials

Ivacaftor and lumacaftor were purchased from Selleck Chemicals (Stratech Scientific Ltd., Newmarket, UK). CFTR_inh_-172, cycloheximide and forskolin were purchased from Sigma-Aldrich (Gillingham, UK), and PKA purified from bovine heart was from Calbiochem (Merck Chemicals Ltd., Nottingham, UK). All other chemicals were of reagent grade and supplied by Sigma-Aldrich.

ATP was dissolved in intracellular solution, forskolin in methanol, and all other reagents in DMSO. Stock solutions were stored at −20 °C with the exception of those of ATP, which were prepared directly before each experiment. Immediately before use, stock solutions were diluted to final concentrations, and, where necessary, the pH of the intracellular solution was readjusted to pH 7.3 to avoid pH-dependent changes in CFTR function (73). Precautions against light-sensitive reactions were observed when using CFTR modulators. DMSO was without effect on CFTR activity (43, 74). Upon completion of experiments, chambers were thoroughly cleaned before reuse (for patch clamp studies, see Ref. 32); for Ussing chamber studies, soaked overnight in 3% (v/v) Mucasol^TM^ (Merz GmbH) diluted in hot water before thorough washing with double-distilled water).

##### Data Analysis

Results are expressed as means ± S.E. of *n* observations. To test for differences between groups of data, we used Student's *t* test or an analysis of variance. Differences were considered statistically significant when *p* < 0.05. All tests were performed using SigmaPlot (Systat Software Inc., Richmond, CA).

## Author Contributions

R. C. F. and D. N. S. conceived and coordinated the study, designed experiments, interpreted data, and wrote the manuscript. X. M. designed, performed, and analyzed the experiments in Figs. 4 and 5, interpreted data, and wrote the manuscript. Y. W. designed, performed, and analyzed the experiments in Figs. 1–3 and 7, interpreted data, and wrote the manuscript. X. W. designed, performed, and analyzed the experiments in Fig. 6 and interpreted data. J. A. W. and H. L. designed, performed, and analyzed the experiments in Fig. 8 and interpreted data. T. L. R. designed and generated the F508del- and G551D-CFTR constructs used in Figs. 4–6. Z. C. contributed the G551D-CFTR data shown in Figs. 1 and 2 and interpreted data. All authors reviewed the results and approved the final version of the manuscript.

## Footnotes

8 The abbreviations used are:

CF: cystic fibrosis
CFTR: cystic fibrosis transmembrane conductance regulator
ABC: ATP-binding cassette
CPM: *N*-[4-(7-diethylamino-4-methyl-3-coumarinyl)phenyl]maleimide
DDM: dodecyl-β-d-maltoside
FRT: Fischer rat thyroid
*I*_sc_: short-circuit current
MSD: membrane-spanning domain
NBD: nucleotide-binding domain
PKA: protein kinase A
*P*_o_: open probability
*P*_o(app)_: apparent open probability
TES: *N*-tris[hydroxymethyl]methyl-2-aminoethanesulfonic acid
*R*_t_: transepithelial resistance.
